# Predictive value of antinuclear antibodies in autoimmune diseases classified by clinical criteria: Analytical study in a specialized health institute, one year follow-up

**DOI:** 10.1016/j.rinim.2013.10.003

**Published:** 2013-11-09

**Authors:** María Elena Soto, Nidia Hernández-Becerril, Ada Claudia Perez-Chiney, Alfredo Hernández-Rizo, José Eduardo Telich-Tarriba, Luis Eduardo Juárez-Orozco, Gabriela Melendez, Rafael Bojalil

**Affiliations:** a Department of Immunology, Instituto Nacional de Cardiología Ignacio Chávez, Mexico City, Mexico; b Universidad Panamericana School of Medicine, Mexico City, Mexico; c Universidad Nacional Autónoma de México, Faculty of Medicine, Mexico City, Mexico; d Department of Cardiovascular Magnetic Resonance, Instituto Nacional de Cardiología Ignacio Chávez, Mexico City, Mexico; e Department of Health Care, Universidad Autónoma Metropolitana-Xochimilco, Mexico City, Mexico

**Keywords:** Antinuclear antibodies, Generalized rheumatic disease, Clinical criteria, Predictive value.

## Abstract

*Introduction*: Determination of antinuclear antibodies (ANA) by indirect immunofluorescence (IIF) is usually the initial test for the diagnosis of systemic rheumatic diseases (SRD). Assigning predictive values to positive and negative results of the test is vital because lack of knowledge about ANAs and their usefulness in classification criteria of SRD leads to inappropriate use. *Methods*: Retrospective study, ANA tests requested by different specialties, correlation to patients' final diagnosis. *Results*: The prevalence of autoimmune disease was relatively low in our population yielding a low PPV and a high NPV for the ANA test. 40% of the patients had no clinical criteria applied prior to test. Coexistence of two or more autoimmune disorders affects prevalence and predictive values. *Conclusion*: Application of the test after careful evaluation for clinical criteria remarkably improves the positive likelihood ratio for the diagnosis.

## 1 Introduction

Immunological assays for the detection of antinuclear antibodies (ANA) are useful and important complementary tools for the diagnosis and follow-up of patients with autoimmune diseases [1]. The identification of the antigen–antibody coupling is the common end-point for all techniques; however, several differences exist as for the utility, sensitivity, specificity, and predictive values of each test [1], [2].

In general, if a patient presents clinical manifestations of an autoimmune disease, the first test to be requested is ANA detection by indirect immunofluorescence using HEp-2 cells, due to its great sensitivity [1], [3]. The different possible patterns, the intensity, and the titers obtained by consecutive dilutions must be carefully examined. Identification of the antigens recognized by the ANA is further evaluated by more specific tests such as ELISA, radioimmunoanalysis (RIA) or electroimmunotransference (EIT) [2], [4].

The use of these tests requires knowledge of their fundamental aspects and also of the clinical classification criteria of each disorder in order to contribute to an appropriate diagnosis [5], [6].

The usefulness of this testing has been evaluated in retrospective studies of patients with systemic rheumatic disease (SRD), and it has been proven that its positive predictive value is low due to the relatively large amount of false positive results. For specific rheumatic diseases, the ANA test yields a positive predictive value of 11%, a negative predictive value of 97%, and a sensitivity and specificity of 42% and 85% respectively [7].

Several physiological and pathological factors might favor the development of ANA in the non-rheumatic population, such as pregnancy, advanced age, family history of autoimmune disease, as well as infectious, cardiovascular or oncological diseases [8], [9], [10], [11], [12]. This situation conveys challenges such as interpretative standardization [13].

A high percentage of patients with high autoantibodies titers do not manifest any clinical signs of autoimmune disease. This may be due to the existence of circulating antigens that are not routinely tested for, such as those resulting from infectious stimuli, from multifactorial synthesis or those naturally produced by CD5+ cells [14]. For this reason, clinicians should pay close attention to the titers in which the ANAs are reported, taking into account that in healthy individuals, antibodies should be negative or can be present in low titers, and that intermediate titers may be present in non-affected relatives of patients with autoimmune diseases or in elders with chronic infections or neoplasms [8], [11], [12], [15].

In Mexico, ANA prevalence has been studied in healthy individuals and consensus has been reached as to consider positive a gross mottled pattern in dilutions over 1:160, while homogeneous, centromeric, peripheral or centriolar patterns should be considered positive even in dilutions as low as 1:40 [16]. Their presence can be, nevertheless, due to natural antigens [14], [17], [18].

In some instances the recognition of antibodies directed to known antigens cannot be achieved. This complicates the accurate measurement of the antibody’s predictive value [19], [20].

The objective of the present study was to determine the predictive values (PPV, NPV) of ANA testing for suspected SDR by analyzing the pre-test assessment of rheumatologic clinical criteria as well as post-test diagnosis.

## 2 Methods

We analyzed samples for ANA studies requested to our lab during a twelve-month period. The tests were selected if they were performed by IIF in HEp-2 cells (INOVA Diagnostics INC San Diego USA) and if an initial positive result at a 1:40 dilution led to successive dilutions. An informed consent was obtained for each test form each patient.

Furthermore, the presence of specific auto-antibodies was evaluated by ELISA (ORGENTEC Diagnostica GmBh Carl-Seiss Mainz,Germany) using purified extractable nuclear antigens (ENA) for Sm, RNP/Sm, SSA/Ro, SSB/LA, Anti-Scl-70, and anti-centromere as well as crithidia luciliae substrate.

An ANA test was considered to be positive when titers were superior to the following dilutions: Nuclear pattern: homogeneous>1:40, coarse speckled and fine speckled>1:160, laminar and peripheral>1:40. Cell cycle: nucleolar, centromeric, and centriolar>1:40. Cytoplasmic>1:80 and micotocondrial>1:160.

Each patient's clinical file was reviewed by a qualified rheumatologist to acknowledge, if the suspected diagnosis was confirmed or if there was an alternative final diagnosis. We confirmed form the records the evaluation made for the presence of diagnostic clinical criteria in each patient. Clinical criteria considered for each disease the following the guidelines for diagnosis.

### 2.1 Statistical analysis

Sample size was calculated by correlation as follows:

$$n=\frac{n\prime }{1+n\prime \cdot N}$$

$$n\prime =\frac{S^{2}}{\sigma^{2}}$$

where *N*=1374, standard error, Se=0.025, *p*=0.18, *S*^2^ the sample variance *p*(1−*p*)=(018)(1−*p*)=(0.18) (0.82)=0.1476}, *σ*^2^ is the population variance, (Se)^2^ (0.025)^2^=0.000625.

$$n\prime \frac{S^{2}}{\sigma^{2}}=\frac{0.1476}{0.000625}=236$$

$$n\frac{n\prime }{1+n\prime /Ν}=\frac{236}{1+236/1374}=\frac{236}{1+0.17176}=\frac{236}{1.17176}=201.5=202$$

**Sample size**=**202.**

We included a total of 373 samples for this study, tendency measures with mean and standard deviation were obtained for variables with parametric distribution. Non-parametric variables were analyzed with percentages, median and ranges. Statistical analysis was performed using Spearman’s correlation, Chi-square and exact Fisher’s test with SPSS version 16 and Epi-info version 6.

## 3 Results

A total of 373 requests for ANA evaluation were received. 299 (80%) corresponded to women and 74 (20%) to men. Mean age was 40±15 and 37±17 years, respectively.

In 364 (83%) samples, nuclear antibodies were found with dilutions 1:40 and no antibodies were found in 9 cases (2%). In 193 out of the 364 (52%) antibodies against specific antigens were found.

Frequency of test requests and use of clinical criteria by each department from our institution are shown in Table 1.

There was a total of 373 ANA tests performed. In 364 (98%) patients, antibodies were found in dilutions of 1:40. Out of these, SRD was confirmed in 213 (57%) cases and it could not be confirmed in 160 (42%).

From the 213 patients with confirmed SRD, in 187 (88%) clinical criteria were applied prior to the blood test, but only in 167 (78%) SRD was confirmed. In 20 patients (9%), the clinical criteria were applied but no autoimmune disease could be diagnosed. 120 individuals (56%) were diagnosed with SRD by both clinical and laboratory criteria. In 47 cases (22%), clinical criteria were used prior to requesting the ANA test but no antigen-specific antibodies were found; 22 (10%) patients had no clinical criteria applied prior to requesting the test, but ANAs could be found in high dilutions and were positive against specific antigens. Therefore the physician concluded that the patients had an autoimmune disease. Finally, there were 24 patients (11%) with no clinical criteria applied prior to soliciting the ANA test and no antigen-specific antibodies were found, however, they presented IIF patterns compatible with autoimmune disease on in high titers.

No autoimmune disease was found in 160 patients. In 6 (4%) of them, clinical criteria were applied prior to soliciting the ANA test, but antibodies were found only in dilutions of 1:40 with antigen-specific antibodies, and in 49 patients (31%) no clinical criteria were applied and the antibodies were found only in low ANA titers and antigen-specific antibodies. Also, from these 160 patients, 91 (57%) had not previously met the mentioned clinical criteria according to the suspected SRD.

These also corresponded mainly to patients with heart or kidney disease.

Dilutions that predominated were 1:40 although there was some percentage of antibodies in high titers. Even an antigen-specific antibody could be found in 8 cases; in these, the attending physicians applied their clinical judgment and discarded SRD. Regardless if they presented specificity towards an antigen or not, when the criteria were applied SRD was demonstrated in 167 patients (78%) while SRD could only be diagnosed in 46 (22%) when no criteria were used.

No SRD was found in 141 (88%) of the requests for ANA test in which no clinical criteria were applied compared to 19 (12%) in the group in which clinical criteria were used. This difference achieved statistical significance with an OR of 26 (95% CI 14–50, *p*<0.0001). This analysis strongly supports the application of clinical criteria prior to the request of antibody testing in patients in whom autoimmune disease is suspected.

The pretest probability of the antigen-specific antibody test is of 57%. Whenever clinical criteria are applied, we observed an improvement of 32%. Nevertheless, when the antibody test was used to confirm SRD without the use of clinical criteria this value diminished to 15% as shown in Table 2.

Further analysis of the information revealed that when clinical criteria and specific antibodies are present, we achieve a sensitivity of 57% and a specificity of 96%; when one or both are negative we get an increase in sensitivity but a loss in specificity. We evaluated the effect of various combinations of these results in Table 3, Table 4

The aforementioned improvement in the post-test likelihood ratio results should be observed every time considering both tools (clinical criteria and specific antinuclear antibody testing) are not independent for the diagnosis of autoimmune disease but usually sequential.

Pre-test probability is 0.57

$$\frac{0.57}{1-0.57}=1.3$$

The likelihood ratio for stand-alone testing and their combined utility is shown in Table 5, in panel A we see the that pre-test likelihood for a patient with suspected autoimmune disease when a test for antigen-specific antibodies is requested is 1.94; the post-test probability with an antigen-specific antibody can be calculated as follows:

$$\frac{1.94}{1.94+1}=65\%\;\;\mathrm{Probabilitypost}-\mathrm{test}$$

Calculating the post-test likelihood ratio when clinical criteria were used to classify the disease we obtain a result of 3.55 (Panel B) and a post-test probability of 78%.

$$\frac{3.55}{3.55+1}=78\%\;\mathrm{Probabilitypost}-\mathrm{test}$$

Panel C shows the result from combining both assessments against the application of only one evaluation (clinical or serological).

As presented in Table 6, Panel A (patients with autoimmune disease were=213), sensitivity was 72% when clinical criteria were used prior to antigen-specific antibodies (T-tasa FP) while it only was 48% when they were not applied and antibody testing was performed directly.

Both tests are independent (in Panel B) (patients without autoimmune disease=160), the specificity for not having autoimmune disease was of 65% when the clinical criteria were negative or not used. However, when the test was negative and clinical criteria had been used, the specificity of ANA was 70% in this series. In patients in whom antigen-specific antibodies are found, the probability to diagnose them by clinical classification criteria is high while patients with a negative antibody test have a higher probability of not presenting any clinical manifestations. This supposed concordance should be further evaluated in larger series.

As shown in Table 7 panel a, the likelihood ratio to find an autoimmune disease is of 14.97 when both clinical criteria and the ANA test for specific antigens yield positive results; a repeated ANA test would not significantly increase the likelihood ratio.

As shown in Table 7 panel b, clinical criteria as a stand-alone tool (LR+=6.24) and the study of ANA against specific antigens (LR+=2.4) when positive results in a high LR of 6.24×2.4=14.97; likewise, the likelihood ratio when both tests are positive, starting form ANA against specific antigens as a stand-alone tool (LR+=1.94) and clinical criteria (LR+=7.72) when positive results in a similar LR, 1.94×7.72=14.97. Therefore, the decision to request an antigen-specific antibody test can become more complex.

In this study we also described the frequency of ANA, specificity for specific antigens, and type of nuclear pattern and their relationship with SRD. These results are shown in Table 8.

Equally rare patterns such as pattern NuMA antibodies were found in 7 cases (2%), out of which 5 were women and 3 men, with a mean age of 33 (28–64) for women and 52 (21–57) for men. Two of them were diagnosed with secondary APS, one with SLE plus AS, another one with SLE plus limited systemic sclerosis, one with rheumatoid arthritis and Sjogren's syndrome, one with ANCA-associated vasculitis and pulmonary thromboembolism, and a last one with atrial tachycardia plus pulmonary arterial hypertension.

Two females presented PCNA antibodies; one was diagnosed with Takayasu arteritis and the other one with SLE plus dilated myocardiopathy. Two cases were reported as having antibodies against proliferating cells.

In non-rheumatic diseases we found anti-DNA antibodies, 3% in patients with cardiopathy, 33% in those with hypothyroidism and 13% in nephropathies not associated with SLE. Predictive values of the tests in relationship with clinical phenotype of SRD are shown in Table 9.

**Fig. 1:**
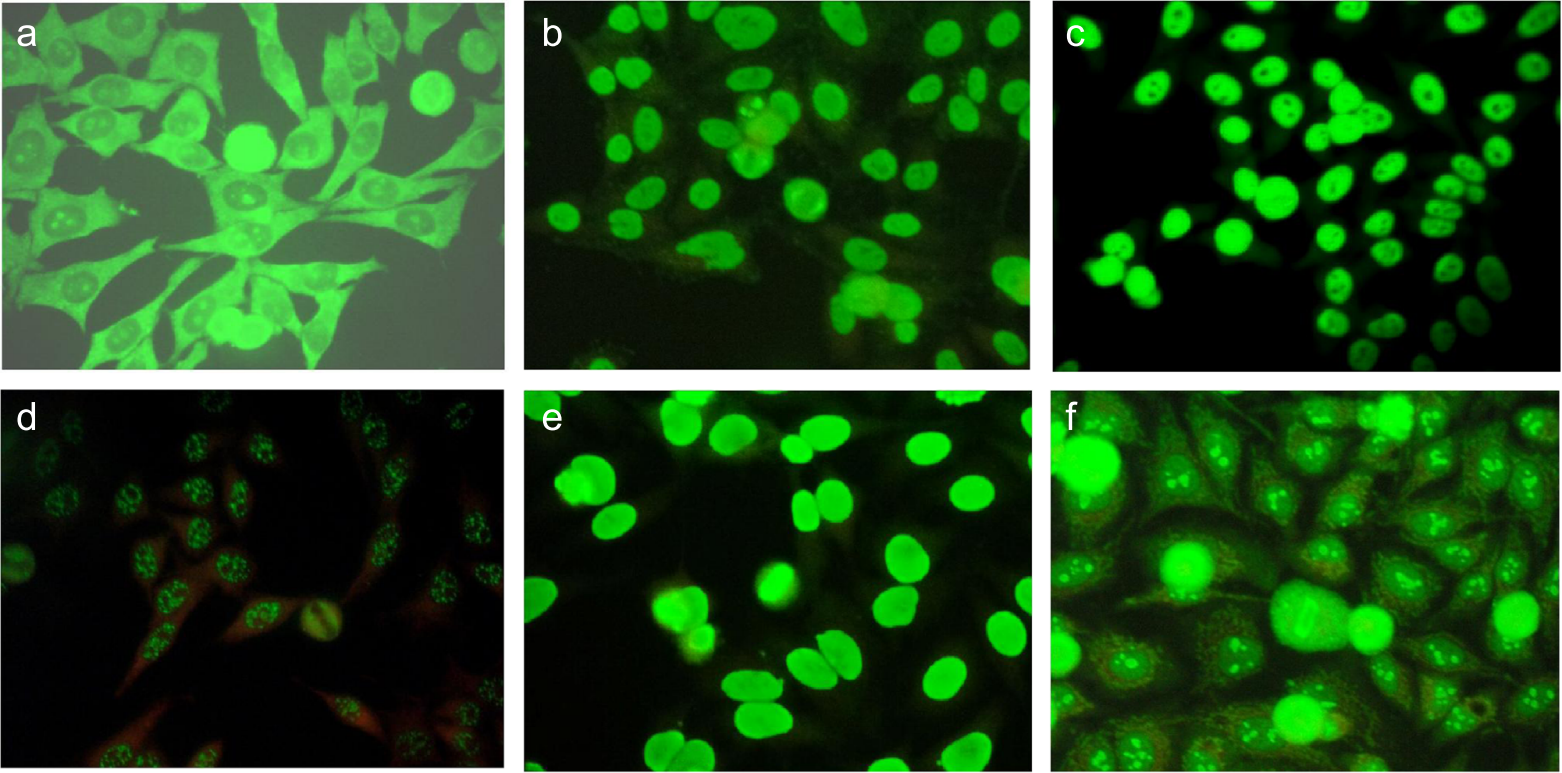
Microphotographs of indirect immunofluorescence of specific antibodies in Hep2 cells. (a) Cytoplasmic P-ribosomal pattern. (b) NuMA-1 pattern. (c) Centromeric or discrete speckled pattern. (d) SSA discrete speckled pattern. (e) Homogeneous pattern. (f) Nucleolar with mitochondrial pattern.

The frequency of different patterns of antibodies documented in this sample were: discrete speckled (DS) 179 (48%), DS- centromere 8 (2%), DS-NuMA 3 (0.8%), DS-Na, DS-Jo, DS-mitochondrial, DS-nucleolar (N) and DS-homogeneous patterns were present in 0.3% each. Homogeneous pattern (H) in 109 (29%), both H and N in 3 (0.8%), H-speckled in 1 (0.3%); Coarse speckled in (CS) 17 (5%), CS-NuMA 4 (1%), speckled 2 (0.5%), cytoplasm 1 (0.3%). Homogeneous, DS and CS patterns were all observed in high titers (Fig. 1).

Thirty-five (22%) patients without autoimmune disease presented ANA in 1:40 dilutions, but none were observed in dilutions over 1:320.

In cases with SRD, ANA could be found in dilutions over 1:320 in 199 (57%) compared to those without autoimmune disease 44 (26%), OR 3.45 (CI 95%, 2.19–5.50), and in dilutions between 1:2560 and 5120 71 (39%) in SLE, scleroderma, mixed connective tissue disease, overlap, Sjogren’s syndrome and rheumatoid arthritis plus systemic lupus erythematous. Even though they were observed in non-autoimmune diseases, this percentage was lower: 12 (8%), OR 5.88 (CI 95%, 2.95–11.94) (Fig. 1a–h).

## 4 Discussion

It is known that positive and negative predictive values for any test are dependent upon the disease's prevalence, with false positive results increasing in those samples in which the disease has a low prevalence, therefore decreasing the test's positive predictive value [7].

Healthy subject, people with non-autoimmune diseases and those with a family history of autoimmune disease present a high percentage of antibodies in low titers [21], [22].

Our series reveals that the predictive value of the test is low, and that it is lower if proper clinical criteria are not applied when requesting the test [21]. Positive antibodies in low titers may lead to confusion when trying to establish a diagnosis, and can become problematic when they are found at higher titers.

It is well known that any test such as antibodies against a specific antigen conveys false positive and false negative results. This can lead to diagnostic and therapeutic errors by utilizing measures when they are not required [23], [24].

In this analysis, we see that using clinical criteria before requesting the test provides a considerable improvement in the diagnostic workup. Antibodies considered to be specific for SLE, such as double strand anti-DNA, have been reported as well in Sjogren's syndrome, dermatomyositis and cutaneous sclerosis [25], [26], [27], [28], [29]. In our series the percentage of SLE patients with positive ANA was of 49%, with varying frequency ranges from 6% to 50%,when SLE coexisted with other diseases, and in 90% of patients with renal damage, a finding known to bear a worse prognosis [30], [31], [32], [33]. In non-rheumatic diseases we found anti-DNA antibodies in frequencies similar to those previously reported in the literature, supporting the idea of the existence of an immunological alteration in cardiovascular and renal diseases, which might be explained by previous infections [15], [34], [35].

Antibodies directed towards ribonucleoproteins (SM, RNP, SSB) are usually detected in SLE, but not in discoid lupus. Our results concur with previous literature [31], [36]. As for SM antibodies, there are reported presence of them in 15–40% of cases; we found that they are present in 30% of cases of SLE when not associated with other diseases, with ranges that vary from 15% to 50% when another SRD coexists with SLE or there is damage to a specific organ [37], [38]. Quite remarkable, elevated ANA titers are important in the diagnostic of rheumatic diseases, but it is also very important to be familiar to each laboratory's cut-off points. Also the type of pattern of antibodies was found in some cases in close correlation with the presence of some autoimmune diseases. It is known that antibodies directed against ribonucleoproteins are associated with connective tissue diseases [39]. An homogeneous pattern might be proof of reaction against native single or double stranded DNA and associated with SLE. The centromeric pattern is characteristic of CREST syndrome and those against nucleolar RNA are associated with SLE and systemic progressive sclerosis [40]. However, other unusual nuclear ANAs are those against the Nuclear mitotic apparatus (NuMA), which might or might not be reported accurately depending upon the laboratory's experience [41]. Their positivity is associated with connective tissue disease, 45% corresponding to Sjogren’s syndrome and undifferentiated connective tissue disease as well as autoimmune diseases against specific organs in 17% even though up to 38% have been found in non-autoimmune diseases [42].

In this study 2% of patients had NuMA, and they were associated with primary AS, one of them with optic neuritis and a possible Devic syndrome. The prevalence of these antibodies and their clinical significance has been previously reported in the literature [43], [44].

Antibodies against the nuclear antigen of proliferative cells were described over 30 years ago in patients with chronic hepatitis B or C, and they have only been found in about 5% of patients with SLE. Their clinical significance has been recently studied in a metanalysis and they have also been detected in polymyositis, systemic sclerosis and even healthy individuals. However their prevalence has not surpassed 2% in any group [45]. In this study, they were detected in two cases, one of them a 64-year-old woman with SLE and end stage renal disease, and the second one in a 23-year-old female with Takayasu arteritis and systemic arterial hypertension.

The presence of specific antibodies against cellular components such as nuclear or cytoplasmic molecules are specific for some diseases [46], [47] while some other might be completely nonspecific [48], [49]. Moreover other findings might depend upon a clinical characteristic of the disease, such as neuropsychiatric lupus in which anti-p ribosomal antibodies have a 10% prevalence and were observed in 2% of all patients with SLE [46]. Nevertheless, some antibodies are related with organ specific alterations and could be prognostic markers [50]. Commonly, when a non-rheumatologist specialist requests an ANA test in a patient it is due to the presence of inflammatory signs and symptoms that most physicians would not overlook. However antibody-testing results does not consider previous clinical details and specific diagnosis becomes quite difficult [51], [52], [53], [54].

We were able to confirm the dispersion and utility that these results have depending upon the clinicians’ specialty, the use of clinical criteria, and indirectly, the knowledge of some recommendations from guidelines.

We believe that in some cases the severity of the clinical picture and diagnostic uncertainty may justify requesting for these tests, however a positive result might turn out to be a confusing factor and therefore require an interpretation that should into account, in first place, the clinical context.

The use of the test in patients with SRD and a positive result might lead to a second test. Several studies attempting to obtain an appropriate use of laboratory tests have been published with the fair purpose of reducing unnecessary testing [4], [55], [56].

A non-medical factor, knowledge of the techniques and standardized procedures, contributes to the optimal use of the test. Other variables could contribute to the variability of the results such as ethnicity, the use of clinical criteria, and the coexistence of several autoimmune diseases or presence of several other antigens.

On the other hand, when the prevalence of a disease in a sample is low, positive predictive value tends to be low as well dictating the need to confirm the result by using a second test.

We found a low prevalence of autoimmune diseases in the requests of these tests, which were solicited by several specialists with differing criteria.

From another view, the high percentage of false positives may be attributed to the fact that only a certain set of antibodies are routinely tested for, not including other recognized antibodies such as antihistone, antinucleosomes, CENP-B, CENP-A, CENP-C, Sp100 protein, PML or NDP53, which could increase the predictive value of the test [57], [58], [59], [60], [61], [62].

Specificity can be increased when clinical criteria and diagnostic algorithms are applied. This reduces unnecessary ANA tests and correlating with a better analysis, utilization, and clinical judgment by the physicians [52], [63], [64].

## 5 Conclusion

Positive and negative predictive value for the ANA test is low, it is dependent on the clinical context of the patient and if the physician relies on clinical criteria for its request. The use of clinical criteria specific for each probable disease prior to antinuclear antibodies testing increases the likelihood ratio for the diagnosis of autoimmune diseases. This also depends upon the phenotype of the disease and the coexistence of two or more diseases or the presence of other antigens, which are not routinely tested for in all laboratories.

The proper use of laboratory tests, in accordance to knowledge and interdisciplinary communication, significantly improves the diagnostic yield of specialized evaluations.

## Figures and Tables

**Table 1:** Percentage of positive ANA requested by several medical specialties and correlation with the use of clinical classification criteria.

Department	Total of tests requested (%)	Used clinical criteria	Antibodies in 1:40 dilution	Did not use clinical criteria	With specific antigen	With confirmed AD 213	With autoimmune disease (AD) 213				Without AD 160	Without autoimmune disease 160			
UCCBR and Sp (+)	UCCBR and Sp (−)	NUCBR and Sp(+)	NUCBR and Sp (−)	UCCBR and Sp (+)	UCCBR and Sp (-)	NUCBR and Sp(+)	NUCBR and (Sp (−)
Rheumatology	278 (75)	170 (61)	274	108 (39)	156	194/278 (70)	111 (57)	44 (23)	18 (9)	21 (11)	84 (30)	4 (5)	11 (13)	25 (30)	44 (52)
Immunology	6 (2)	3 (50)	34	3 (50)	3	5 /6 (83)	2 (40)	1 (20)	1 (20)	1 (20)	1 (17)	0	0	0	1 (100)
Nephrology	34 (9)	9 (27)	18	25 (73)	17	11/34 (33)	7 (67)	1 (9)	2 (18)	1 (9)	23 (68)	0	1 (4)	7 (30)	15 (65)
Adult cardiology	27 (7)	4 (15)	26	23 (85)	8	1/27 (4)	0 (0)	0	1 (100)	0	26 (96)	2 (8)	2 (8)	7 (27)	15 (58)
Cardiopneumology	20 (5)	1 (5)	3	19 (95)	3	2/20 (5)	0	1 (1)	0	1 (50)	18 (90)	0	0	4 (22)	14 (77)
Emergency department	3 (0.8)	0 (0)	4	3 (100)	3	0 (0)	0 (0)	0	0	0	3 (100)	0	0	3 (100)	0
Pediatric cardiology	5 (1)	0 (0)	5	5 (100)	3	0 (0)	0 (0)	0	0	0	5 (100)	0	0	3 (60)	2 (40)
Total	373 (100)	187 (50)	364	186 (50)	193	213/373 (57)	120/213 (56)	47/213 (22)	22/213 (10)	24/213 (11)	160/373 (43)	6/160 (4)	14/160 (8)	49/160 (31)	91/160 (57)

AD: autoimmune disease,UCCBR: utilized clinical criteria before the request of test, NUCBR: non-utilized clinical criteria before request of test, Sp(+): positive antibody specificity Sp(−): negative antibody specificity.

**Table 2:** Systemic rheumatic disease probability prior to test antibodies.

Antibodies to specific antigen	With autoimmune disease	Without Autoimmune disease		Pretest probability (specific antigen)	Change of the probability after the test (%)
+	142	55	197	142/197=72% (PPV)	**15**
−	71	105	176	105/176=60% (NPV)	**3**
Total	213	160	373		
	*S*=(142/213)=67%	*E*=(105)/(160)=66%			

Solicitors utilization of clinical criteria	With autoimmune disease	Without autoimmune disease		Pretest probability (clinical criteria)	Change of the probability after the test (%)

+	167	20	187	167/187=89%	**32**
−	46	140	186	140/186=75%	**18**
Total	213	160	373		
	*S*=167/213=78%	*E*=140/160=88%			
					

The application of clinical criteria appropriate to request the test is 213/373=57%.

*S*=sensibility, *E*=specificity.

**Table 3:** Complex tables to evaluate both tools for diagnosis of autoimmune diseases (appropriate clinical criteria and specific auto-antibodies).

Clinical criteria and specific antibodies	With autoimmune disease	Without autoimmune disease	
Both positive	120	6	127
One positive	69	63	130
Both negative	24	91	116
Total	213	160	373


Effect of locating the cut-off point at different levels			
Between one and both positive (WCC) (Sp+antibodies)		Between one and both negative	
120	6	189	69
93	154	24	91
213	160	213	160
*S*=57%	*E*=96%	*S*=89%	*E*=57%

*S*=sensitivity, *E*=specificity.

**Table 4:** Complex tables to evaluate both tools for diagnosis of autoimmune diseases (appropriate clinical criteria and specific auto-antibodies).

Cut-off point between one and two positive tests	With autoimmune disease	Without autoimmune disease		Likelihood of autoimmune disease after the test
Both positive test (clinical criteria+ specific antibodies)	120	6	127	120/127=94%
One or both negative	93	154	246	93/246=38%
Total	213	160	373	


Cut-off point between one and two negative tests	With autoimmune disease	Without autoimmune disease		Cut-off point between one and both tests negative
One or both positives	189	69	258	189/258=73%
Both clinical criteria and specific antibodies negative	24	91	115	24/115=21%
Total	213	160	373	

**Table 5:** Likelihood ratio for each test.

**(a) Specific-antigen antibodies**					
Antibodies for specific antigen	With autoimmune disease		Without autoimmune disease		Likelihoood ratio
Number	Proportion	Number	Proportion
+	142	142/213=0.66	55	55/(160=0.34	0.66/(0.34=1.94
−	71	71/230=0.30	105	105/160=0.65	0.30/0.65=0.46
Total	213		160		

**(b) Clinical criteria**					
Classification according to criteria	With autoimmune disease		Without autoimmune disease		Likelihood ratio
Number	Proportion	Number	Proportion

+	167	167/213=0.78	20	20/160=0.34	0.78/0.22=3.55
−	46	46/213=0.22	140	140/160=0.88	0.30/0.65=0.39
Total	213		160		

**(c) Effect of different combinations of specific antibodies and clinical criteria over the diagnostic workup for SRD**					
	Number	Proportion	Number	Proportion	Likelihood ratio

Clinical criteria and specific antibodies positive	120	120/213=0.56	6	6/160=0.037	0.56/0.037=15.02
One or both negative	93	93/213=0.44	154	154/160=0.96	0.44/0.96=0.45
Total	213		160		

**Table 6:** Evaluation of the combination of positive results on specific antibodies and utilization of clinical criteria for the diagnosis of autoimmune disease.

**Panel A: Patients with autoimmune disease**						
	Positive specific antigen antibodies			Sensitivity of specific antigen antibodies (false positive ratio)		
Positive	Negative
With clinical criteria	120	47	167	With clinical criteria (+)		120/167=72%
Without clinical criteria	22	24	46	Without clinical criteria (−)		22/46=48%
Total	142	71	213	160		142/213=67%

	Sensitivity of specific antigen antibodies (1-true positive ratio)					
When specific antibodies (+)	When specific antibodies (−)	Total

	120/142=85%	47/71=66%	167/213=78%			


**Panel B: Without autoimmune disease**						
	Positive specific antigen antibodies			Sensitivity of specific antigen antibodies (false positive ratio)		
Positive	Negative
With clinical criteria	6	14	20	With clinical criteria (+)		14/20=70%
Without clinical criteria	49	91	140	Without clinical criteria (−)		91/140=65%
Total	55	105	160	160		105/160=65%

	Sensitivity of specific antigen antibodies (1-true positive ratio)					
When specific antibodies (+)	When specific antibodies (−)	total

	49/55=89%	91/105=87%	140/160=87%			

**Table 7:** (a) Likehood ratios when two converging tools for autoimmune disease are positive. (b) Likelihood ratios for SRD diagnosis.

(a)			
	Autoimmune disease		Likelihood ratio
Present	Absent
Both positive tool (clinical criteria CC and specific ANA)	120 (56%)	6 (3.7%)	0.56/0.038=14.97
0.44/0.96=0.46
One or both	47	14	
+22	+49
+24	+91
–	–
93 (44%)	154 (96%)
Total	213 (100%)	160 (100%	

(b)			
	Positive Likelihood Ratio (L.R.+)		
Clinical criteria	AAN against specific antigen	

Stand-alone test	1−(0.78/(1−0.87))=6.24	0.66/(1−0.66)=1.94	
When the other test was positive	(0.85/(1−0.89))=7.72	0.72/(1−0.70)=2.4	

**Table 8:** Frequency of nuclear patterns and antibodies against specific antigens in several autoimmune diseases.

Disease	Antigen						Pattern					
n	SM	RNP	SSA	SSB	SCL70	Anti-centromere	Homogeneous	Discrete speckled	Coarse speckled	NUMA	PCNA
SLE	43	13 (31)	13 (30)	13 (30)	4 (9)	1 (2)	1(2)	24 (56)	13 (27)	0	0	1 (2)
RA+SLE	8	2 (10)	3 (38)	4(50)	2 (25)	0	0	5 (63)	6 (75)	0	0	0
SLE+APS	13	1 (5)	2 (15)	4 (30)	0	0	0	4 (31)	6 (43)	0	1 (8)	0
SLE+SS	3	0	1 (33)	2 (67)	2 (67)	0	0	1 (33)	2 (67)	0	0	0
Discoid lupus	6	0	1 (17)	0	0	0	1	2 (33)	2 (33)	0	0	0
SLE+hyperthyroidism	2	0	1(50)	0	0	0	0	1 (50)	3 (50)	0	0	0
SLE+hypothyroidism	5	0	0	3 (60)	1 (20)	0	0	2 (50)	0	0	0	0
RA	17	0	0	1 (6)	0	0	0	10 (59)	2 (12)	0	0	0
RA+SS	10	1 (10)	1 (10)	5 (50)	4 (40)		0	3 (30)	5 (50)	0	1 (10)	0
RA+hypothyroidism	1	0	0	0	0	0	0	1 (100)	0	0	0	0
IJA	5	0	0	0	0	0	0	3 (60)	2 (40)	0	0	0
Scleroderma	16	2 (10)	5 (31)	3(18)	0	1 (6)	3 (19)	1 (6)	7 (44)	0	1 (6)	0
SCL+SS	2	0	0	1 (50)	1 (50)	0	1 (50)	0	1 (50)	0	0	0
SCL	2	0	0	0	0	1 (50)	1(50)	1 (50)	1 (50)	0	0	0
MCTD	5	0	4 (90)	2 (40)	0	1 (14)	0	0	5 (100)	0	0	0
Overlap	8	1 (5)	2 (25)	2 (25)	0	1 (15)	1(15)	3 (38)	0	0	0	0
Polymyositis	2	0	0	1 (50)	0	0	1 (50)	0	0	1	0	0
Dermatomyositis	1	0	0	0	0	0	0	0	1 (100)	0	0	0
SS	8	0	0	0	5 (62)	0	0	2 (25)	5 (63)	0	0	0
SS+hypothyroidism	2	0	0	0	0	0	0	0	1 (50)	0	0	0
Devic syndrome	3	0	0	1 (33)	0	0	0	0	2 (67)	0	0	0
PAPS	38	0	0	2(5)	0	0	1	9 (24)	22 (58)	2 (5)	2 (5)	0
Fibromyalgia	7	0	1(3)	0	0	0	1	1 (14)	4 (57)	0	0	0
Cardiopathy	94	1	5	1	1	0	0	18 (19)	58 (62)	6 (6)	2 (2)	0
Hypothyroidismo	3	0	0	1(3)	0	0	0	2 (67)	1 (33)	0	0	0
Nephropathy	24	0	0	0	0	0	0	3 (13)	9 (38)	4 (17)	0	0
Cancer	2	0	0	0	0	0	0	0	1 (50)	0	0	0
Takayasu’s arteritis	1	0	0	0	0	0	0	0	0	0	0	1

APS=antiphospholipid syndrome, IJA=idiopathic juvenile arthritis, MCTD=mixed connective tissue disease, PAPS=primary antiphospholipid syndrome, RA=rheumatoid arthritis, SCL=scleroderma, SLE=systemic erythematous lupus, SS=systemic scleroderma.

**Table 9:** Sensibility, specificity, and predictive values according to autoimmunity disease and specificity of antibodfies.

	SM	DNA	SSA	SSB	RNP
SLE	*S*=35	*S*=62	*S*=40	*S*=25	*S*=43
*N*=37	*E*=99	*E*=99	*E*=96	*E*=99	*E*=87
	PPV=93	PPV=96	PPV=70	PPV=91	PPV=71
	NPV=86	NPV=88	NPV=86	NPV=84	NPV=87

RA+SLE	*S*=25	*S*=50	*S*=50	*S*=25	*S*=38
*N*=8	*E*=99	*E*=99	*E*=94	*E*=99	*E*=96
	PPV=67	PPV=80	PPV=31	PPV=67	PPV=30
	NPV=96	NPV=97	NPV=97	NPV=96	NPV=97

Discoid lupus	*S*=0	*S*=20	*S*=0	*S*=0	*S*=25
*N*=6	*E*=94	*E*=99	*E*=94	*E*=99	*E*=96
	PPV=0	PPV=50	PPV=0	PPV=0	PPV=13
	NPV=98	NPV=97	NPV=96	NPV=97	NPV=98

SLE+APS	*S*=10	*S*=17	*S*=33	*S*=0	S=15
*N*=11	*E*=99	*E*=99	*E*=94	*E*=93	*E*=96
	PPV=50	PPV=67	PPV=30	PPV=0	PPV=22
	NPV=94	NPV=94	NPV=95	NPV=93	NPV=93

SLE+SS	*S*=33	*S*=33	*S*=67	*S*=67	S=33
*N*=3	*E*=99	*E*=99	*E*=94	*E*=99	*E*=96
	PPV=50	PPV=50	PPV=18	PPV=67	PPV=14
	NPV=99	NPV=99	NPV=99	NPV=99	NPV=99

SLE+hypothyroidism	*S*=0	*S*=25	*S*=75	S=33	*S*=0
*N*=4	*E*=99	*E*=99	*E*=94	*E*=99	*E*=96
	PPV=0	PPV=50	PPV=25	PPV=50	PPV=0
	NPV=97	NPV=98	NPV=79	NPV=99	NPV=98

Sjogren’s syndrome	*S*=0	*S*=0	*S*=0	*S*=38	*S*=0
*N*=8	*E*=99	*E*=99	*E*=94	*E*=97	*E*=96
	PPV=0	PPV=0	PPV=0	PPV=75	PPV=0
	NPV=99	NPV=96	NPV=99	NPV=97	NPV=95

PAPS	*S*=0	*S*=15	*S*=5	*S*=0	*S*=0
*N*=35	*E*=99	*E*=99	*E*=82	*E*=82	*E*=95
	PPV=0	PPV=80	PPV=18	PPV=0	PPV=0
	NPV=80	NPV=85	NPV=82	NPV=82	NPV=82

RA	*S*=0	*S*=20	*S*=13	*S*=0	*S*=0
*N*=16	*E*=99	*E*=99	*E*=94	*E*=99	*E*=95
	PPV=0	PPV=50	PPV=18	PPV=0	PPV=0
	NPV=99	NPV=97	NPV=92	NPV=91	NPV=91

IJA	*S*=0	*S*=20	*S*=0	*S*=0	*S*=0
*N*=5	*E*=99	*E*=99	*E*=94	*E*=99	*E*=95
	PPV=0	PPV=50	PPV=0	PPV=0	PPV=0
	NPV=99	NPV=97	NPV=99	NPV=97	NPV=91

Scleroderma	*S*=67	*S*=13	*S*=23	*S*=0	*S*=38
*N*=12	*E*=99	*E*=99	*E*=95	*E*=99	*E*=96
	PPV=67	PPV=50	PPV=22	PPV=0	PPV=41
	NPV=93	NPV=95	NPV=94	NPV=99	NPV=95

Crest	*S*=0	*S*=0	*S*=0	*S*=0	*S*=60
*N*=5	*E*=99	*E*=99	*E*=99	*E*=99	*E*=96
	PPV=0	PPV=0	PPV=0	PPV=0	PPV=30
	NPV=80	NPV=99	NPV=99	NPV=99	NPV=99

APS=antiphospholipid syndrome, IJA=Idiopathic juvenile arthitis, MCTD=mixed connective tissue disease, PAPS=primary antiphospholipid syndrome, RA=rheumatoid arthritis, SCL=scleroderma, SLE=systemic lupus erythematosus, SS=systemic scleroderma, *S*=sensitivity, *E*=specificity, PPV=positive predictive value, NPV=negative predictive value.

## References

[1] Hernandez Ramirez D.F., Cabiedes J. (2010). Immunological techniques that support the diagnosis of the autoimmune diseases. Reumatol. Clin..

[2] Cabiedes J., Nunez-Alvarez C.A. (2010). Antinuclear antibodies. Reumatol. Clin..

[3] Briolay J., Gioud M., Monier J.C. (1989). Antinuclear antibodies detected by indirect immunofluorescence on HEp2 cells and by immunoblotting in patients with systemic sclerosis. Autoimmunity.

[4] Sheldon J. (2004). Laboratory testing in autoimmune rheumatic diseases. Best Pract. Res. Clin. Rheumatol..

[5] Binder S.R. (2006). Autoantibody detection using multiplex technologies. Lupus.

[6] McBride J.D., Gabriel F.G., Fordham J. (2008). Screening autoantibody profiles in systemic rheumatic disease with a diagnostic protein microarray that uses a filtration-assisted nanodot array luminometric immunoassay (NALIA). Clin. Chem..

[7] Slater C.A., Davis R.B., Shmerling R.H. (1996). Antinuclear antibody testing. A study of clinical utility. Arch. Intern. Med..

[8] Kiuttu J., Hartikainen A.L., Makitalo R., Ruuska P. (1994). The outcome of pregnancy in antinuclear antibody-positive women. Gynecol. Obstet. Invest..

[9] Kulthanan K., Jiamton S., Omcharoen V., Linpiyawan R., Ruangpeerakul J., Sivayathorn A. (2002). Autoimmune and rheumatic manifestations and antinuclear antibody study in HIV-infected Thai patients. Int. J. Dermatol..

[10] Martinez-Cordero E., Bessudo-Babani A., Trevino-Perez S.C., Teran L., Selman M., Martinez-Miranda E. (1989). Circulating autoantibodies in patients with pigeon breeder's disease. Allergol. Immunopathol..

[11] Ward M.M. (1998). Laboratory testing for systemic rheumatic diseases. Postgrad. Med..

[12] Xavier R.M., Yamauchi Y., Nakamura M. (1995). Antinuclear antibodies in healthy aging people: a prospective study. Mech. Ageing Dev..

[13] Satoh M., Chan E.K., Sobel E.S. (2007). Clinical implication of autoantibodies in patients with systemic rheumatic diseases. Expert Rev. Clin. Immunol..

[14] Abu-Shakra M., Shoenfeld Y. (2007). Natural hidden autoantibodies. Isr. Med. Assoc. J..

[15] Fernandez-Madrid F., Mattioli M. (1976). Antinuclear antibodies (ANA): immunologic and clinical significance. Semin. Arthritis Rheumatol..

[16] Marin G.G., Cardiel M.H., Cornejo H., Viveros M.E. (2009). Prevalence of antinuclear antibodies in 3 groups of healthy individuals: blood donors, hospital personnel, and relatives of patients with autoimmune diseases. J. Clin. Rheumatol..

[17] George J., Gilburd B., Shoenfeld Y. (1997). The emerging concept of pathogenic natural autoantibodies. Hum. Antibodies.

[18] Baumgarth N., Tung J.W., Herzenberg L.A. (2005). Inherent specificities in natural antibodies: a key to immune defense against pathogen invasion. Semin. Immunopathol..

[19] Hamaguchi Y. (2010). Autoantibody profiles in systemic sclerosis: predictive value for clinical evaluation and prognosis. J. Dermatol..

[20] Verstegen G., Duyck M.C., Meeus P., Ravelingien I., De V.K. (2009). Detection and identification of antinuclear antibodies (ANA) in a large community hospital. Acta Clin. Belg..

[21] Hayashi N., Koshiba M., Nishimura K. (2008). Prevalence of disease-specific antinuclear antibodies in general population: estimates from annual physical examinations of residents of a small town over a 5-year period. Mod. Rheumatol..

[22] Sebastian W., Roy A., Kini U., Mullick S. (2010). Correlation of antinuclear antibody immunofluorescence patterns with immune profile using line immunoassay in the Indian scenario. Indian J. Pathol. Microbiol..

[23] Lora P.S., Laurino C.C., Becker B.S., Monticielo O.A., Brenol J.C., Xavier R.M. (2011). Clinical diagnostic performance of different methods for the detection of antibodies to extractable nuclear antigens in connective tissue diseases: a cohort study. Clin. Lab.

[24] Pittock S.J., Lennon V.A., de S.J. (2008). Neuromyelitis optica and non organ-specific autoimmunity. Arch. Neurol..

[25] Buskila D., Berezin M., Gur H. (1995). Autoantibody profile in the sera of women with hyperprolactinemia. J. Autoimmun..

[26] Falanga V., Medsger T.A., Reichlin M. (1987). Antinuclear and anti-single-stranded DNA antibodies in morphea and generalized morphea. Arch. Dermatol..

[27] Gripenberg M., Helve T., Kurki P. (1985). Profiles of antibodies to histones, DNA and IgG in patients with systemic rheumatic diseases determined by ELISA. J. Rheumatol..

[28] Krawiec P., Batko B., Skura A. (2006). Difficulties in differential diagnosis of Sjogren's syndrome and systemic lupus erythematosus. Przegl. Lek..

[29] Warner N.Z., Greidinger E.L. (2004). Patients with antibodies to both PmScl and dsDNA. J. Rheumatol..

[30] Gulko P.S., Reveille J.D., Koopman W.J., Burgard S.L., Bartolucci A.A., Alarcon G.S. (1994). Survival impact of autoantibodies in systemic lupus erythematosus. J. Rheumatol..

[31] Provost T.T., Watson R. (1989). Antinuclear antibodies in systemic lupus erythematosus. Immunol. Ser..

[32] Tan E.M. (1982). Special antibodies for the study of systemic lupus erythematosus: an analysis. Arthritis Rheumatol..

[33] Thompson D., Juby A., Davis P. (1993). The clinical significance of autoantibody profiles in patients with systemic lupus erythematosus. Lupus.

[34] Liang K.P., Kremers H.M., Crowson C.S. (2009). Autoantibodies and the risk of cardiovascular events. J. Rheumatol..

[35] Tonietti G., Oldstone M.B., Dixon F.J. (1970). The effect of induced chronic viral infections on the immunologic diseases of New Zealand mice. J. Exp. Med..

[36] Simmons-O'Brien E., Chen S., Watson R. (1995). One hundred anti-Ro (SS-A) antibody positive patients: a 10-year follow-up. Medicine.

[37] Lussiez V., Combe B., Graafland H., Rucheton M., Sany J. (1989). Anti-Sm and anti-RNP antibodies detected by immunoblotting in disseminated lupus erythematosus. Rev. Rhum. Mal. Osteoartic..

[38] Tan E.M., Chan E.K., Sullivan K.F., Rubin R.L. (1988). Antinuclear antibodies (ANAs): diagnostically specific immune markers and clues toward the understanding of systemic autoimmunity. Clin. Immunol. Immunopathol..

[39] Malleson P.N., Sailer M., Mackinnon M.J. (1997). Usefulness of antinuclear antibody testing to screen for rheumatic diseases. Arch. Dis. Child.

[40] Bernstein R.M., Steigerwald J.C., Tan E.M. (1982). Association of antinuclear and antinucleolar antibodies in progressive systemic sclerosis. Clin. Exp. Immunol..

[41] Andrade L.E., Chan E.K., Peebles C.L., Tan E.M. (1996). Two major autoantigen–antibody systems of the mitotic spindle apparatus. Arthritis Rheumatol..

[42] Mozo L., Gutierrez C., Gomez J. (2008). Antibodies to mitotic spindle apparatus: clinical significance of NuMA and HsEg5 autoantibodies. J. Clin. Immunol..

[43] Bonaci-Nikolic B., Andrejevic S., Bukilica M., Urosevic I., Nikolic M. (2006). Autoantibodies to mitotic apparatus: association with other autoantibodies and their clinical significance. J. Clin. Immunol..

[44] Grypiotis P., Ruffatti A., Tonello M. (2002). Clinical significance of fluoroscopic patterns specific for the mitotic spindle in patients with rheumatic diseases. Reumatismo.

[45] Mahler M., Miyachi K., Peebles C., Fritzler M.J. (2012). The clinical significance of autoantibodies to the proliferating cell nuclear antigen (PCNA). Autoimmun. Rev..

[46] Karassa F.B., Afeltra A., Ambrozic A. (2006). Accuracy of anti-ribosomal P protein antibody testing for the diagnosis of neuropsychiatric systemic lupus erythematosus: an international meta-analysis. Arthritis Rheumatol..

[47] Nozawa K., Fritzler M.J., Chan E.K. (2005). Unique and shared features of Golgi complex autoantigens. Autoimmun. Rev..

[48] Kuwana M., Kaburaki J., Mimori T., Tojo T., Homma M. (1993). Autoantibody reactive with three classes of RNA polymerases in sera from patients with systemic sclerosis. J. Clin. Invest..

[49] Satoh M., Ajmani A.K., Ogasawara T. (1994). Autoantibodies to RNA polymerase II are common in systemic lupus erythematosus and overlap syndrome. Specific recognition of the phosphorylated (IIO) form by a subset of human sera. J. Clin. Invest..

[50] Steen V.D. (2008). The many faces of scleroderma. Rheum. Dis. Clin. N. Am..

[51] Tampoia M., Brescia V., Fontana A., Zucano A., Morrone L.F., Pansini N. (2007). Application of a combined protocol for rational request and utilization of antibody assays improves clinical diagnostic efficacy in autoimmune rheumatic disease. Arch. Pathol. Lab Med..

[52] Damoiseaux J.G., Tervaert J.W. (2006). From ANA to ENA: how to proceed?. Autoimmun. Rev..

[53] Tozzoli R., Bizzaro N., Tonutti E. (2002). Guidelines for the laboratory use of autoantibody tests in the diagnosis and monitoring of autoimmune rheumatic diseases. Am. J. Clin. Pathol..

[54] Solomon D.H., Kavanaugh A.J., Schur P.H. (2002). Evidence-based guidelines for the use of immunologic tests: antinuclear antibody testing. Arthritis Rheumatol..

[55] van W.C., Naylor C.D. (1998). Do we know what inappropriate laboratory utilization is? A systematic review of laboratory clinical audits. J. Am. Med. Assoc..

[56] Solomon D.H., Hashimoto H., Daltroy L., Liang M.H. (1998). Techniques to improve physicians' use of diagnostic tests: a new conceptual framework. J. Am. Med. Assoc..

[57] Ahlin E., Mathsson L., Eloranta M.L. (2012). Autoantibodies associated with RNA are more enriched than anti-dsDNA antibodies in circulating immune complexes in SLE. Lupus.

[58] Koivula M.K., Heliovaara M., Rissanen H. (2012). Antibodies binding to citrullinated telopeptides of type I and type II collagens and to mutated citrullinated vimentin synergistically predict the development of seropositive rheumatoid arthritis. Ann. Rheum. Dis..

[59] Van den B.K., Vercammen M., Regenass S. (2012). Betaine homocysteine methyl transferase 1, a novel auto-antigen associated with anti-Golgi immune reactivity. Clin. Chim. Acta.

[60] Hung W.T., Chen Y.M., Lan J.L. (2011). Antinucleosome antibodies as a potential biomarker for the evaluation of renal pathological activity in patients with proliferative lupus nephritis. Lupus.

[61] Granito A., Muratori P., Quarneti C., Pappas G., Cicola R., Muratori L. (2012). Antinuclear antibodies as ancillary markers in primary biliary cirrhosis. Expert Rev. Mol. Diagn..

[62] Hudson M., Mahler M., Pope J. (2012). Clinical correlates of CENP-A and CENP-B antibodies in a large cohort of patients with systemic sclerosis. J. Rheumatol..

[63] Bonaguri C., Melegari A., Ballabio A. (2011). Italian multicentre study for application of a diagnostic algorithm in autoantibody testing for autoimmune rheumatic disease: conclusive results. Autoimmun. Rev..

[64] Bizzaro N., Tozzoli R., Tonutti E. (1998). Variability between methods to determine ANA, anti-dsDNA and anti-ENA autoantibodies: a collaborative study with the biomedical industry. J. Immunol. Methods.

